# When reintegration fails: Stigmatization drives the ongoing violence of ex‐combatants in Eastern Democratic Republic of the Congo

**DOI:** 10.1002/brb3.2156

**Published:** 2021-05-04

**Authors:** Sabine Schmitt, Katy Robjant, Anke Koebach

**Affiliations:** ^1^ Department of Psychology University of Konstanz Konstanz Germany; ^2^ Non‐Governmental Organization Vivo International Konstanz Germany

**Keywords:** aggression, mental health, Military, social integration, stigmatization

## Abstract

Reintegration of ex‐combatants involves multiple challenges. In addition to the trauma‐related psychological sequelae, social obstacles in the community can aggravate psychopathological aggressive tendencies and lead to the continuation of violence in civilian life. However, the association between others’ negative attitudes and ex‐combatants’ ongoing perpetration of violence remains largely unexplored. Between September 2018 and May 2019, we assessed a representative community sample of adults in Eastern DR Congo (*N* = 1,058) and measured trauma exposure, perpetration, mental health problems (PTSD, depression, and appetitive aggression), perceived stigma (shame, perceived lack of social acknowledgement), experienced stigma, and skepticism toward reintegration with ex‐combatants. Male ex‐combatants (12%, *n* = 129) had more past trauma and violence perpetration than other community members and a greater number of recent conflicts (including both victimization and perpetration) within the community and with strangers/organized violence. They reported more experienced stigma, more severe PTSD symptoms but were less skeptical about reintegration. Ex‐combatants’ ongoing violence was predicted by an interplay of the community's skepticism toward reintegration and ex‐combatants’ perceived and recently experienced stigma (often attributed to the armed group history) and mental health problems, in addition to lifetime traumatization. These findings promote the need for combined interventions that address individual mental health problems including aggression and collective discriminatory attitudes and behaviors.

## Graphical abstract

This article showed that stigmatization presents as additional burden for ex‐combatants in Eastern DRC beyond posttraumatic experiences. Rejective attitudes toward ex‐combatants form the basis of stigmatization and by predicting both mental health problems and aggression present as social drivers of the cycles of violence. Our study demonstrates the importance for intervention programming that combines individual treatment with interventions including ex‐combatants' social environment.

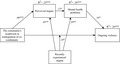

## 1 INTRODUCTION

Interpersonal victimization predicts violent behavior later in life (Johnson et al., 2016; Peltonen et al., 2020; Webb et al., 2017). Moreover, living in a highly aggressive environment can increase the likelihood of adopting violent behavior strategies (Bond & Bushman, 2017; Huesmann & Kirwil, 2007).

In areas of conflict and organized violence, the estimated prevalence of interpersonal violence is particularly high (Fearon & Hoeffler, 2018). The Democratic Republic of Congo (DRC) is the fifth most fragile country in the world (Fund for Peace, 2019). Despite official peace agreements and the presence of the United Nations, fighting continues and hundreds of lives are lost each year (Kivu Security Tracker, 2019). At least 70 armed groups are assumed to be operating in the Kivu regions (Stearns & Vogel, 2015), and there is a high prevalence of ex‐combatants among civilians (about 21% of adults and 10% of youth, Johnson et al., 2010; Mels et al., 2009). After forced (or quasi‐voluntary) enrollment, combatants fulfill a myriad of roles including soldiers, escorts, or auxiliary service providers (e.g., cooks, porters, messengers, or administrators, Elbert et al., 2013). However, sooner or later almost everyone becomes both victim and perpetrator of severe violence (e.g., rape, torture, or murder, Elbert et al., 2013; Robjant et al., 2020a).

### 1.1 Psychological sequelae of armed group life

After military service, combatants often present with various mental health problems including posttraumatic stress disorder (PTSD), depression, suicidality, and substance use disorder (Betancourt et al., 2013; Johnson et al., 2008, 2010; Odenwald et al., 2009; Pompili et al., 2013) as well as heightened levels of aggression (Koebach et al., 2015; Nandi et al., 2017; Weierstall et al., 2013).

Elbert et al. (2010) postulated that after committing severe violent acts, perpetrators can develop an “appetite” for violence and start enjoying the dominance, power, and finally the mere exertion of violence. Appetitive aggression has been reported in various populations including veterans from Germany (Weierstall et al., 2012), Columbia (Weierstall et al., 2013), and Sub‐Saharan Africa (Crombach et al., 2013; Elbert et al., 2010; Nandi et al., 2020) including Eastern DRC (Koebach et al., 2015; Robjant et al., 2019). Cognitive theories of traumatic stress converge in their assumption of a dysfunctional memory (Brewin & Holmes, 2003). Unconsolidated mnesic elements of traumatic events remain detached from their spatiotemporal context (time, space) and form strong mutually excitatory connections (Brewin, 2011). The resulting associative network is often referred as *fear network* (Elbert & Schauer, 2002, 2014). Environmental cues captured by this network can trigger the activation of multiple posttraumatic experiences and cause upsetting intrusions, nightmares, and other symptoms (Elbert & Schauer, 2002). Given the equivalent endocrine reactions that act on and affect the memory during trauma and perpetration, Elbert et al. (2010) drew an analogy of their mnesic structure: after (repeated) perpetration of severe violence, implicit cues of perpetrated acts form excitatory connections bound to positive emotions (lust, pleasure, fascination, and power), giving rise to a hunting associative network. The *hunting network* overlaps with the fear network but emotionally triggers an opposite valence. For example, fast heartbeat or respiration, the sight of blood or the sound of screams and bullets can constitute elements of both victimization and perpetration but may either trigger fear and anxiety (action disposition: flight, submission, dissociation) or excitement and power (action disposition: fight, domination) depending on the dominant neural associations.

Appetitive aggression does not necessarily represent a pathological construct. Rather, it can be understood as adaptation to a highly violent environment in which aggression and violence increase social status (Crombach et al., 2013; Hermenau et al., 2013) and enhance chances of survival (Weierstall et al., 2013). Indeed, a desire for violence likely increases combatants’ functionality by “protecting” against fear and PTSD (Weierstall & Elbert, 2011; Weierstall et al., 2011), at least up to a certain threshold of exposure to traumatic stressors (Hecker et al., 2013; Weierstall et al., 2013). In consequence, violent acts are carried out not only to satisfy extrinsic motivations (e.g., gain of money, food, or drugs) but also because they have become intrinsically rewarding by themselves (Haer et al., 2017). However, after leaving the armed group, this desire for violence becomes maladaptive (assuming a safe postconflict society) and counters the adaptation to non‐ (or less) violent civilian life (Nandi et al., 2017; Robjant et al., 2019). Heightened risks of both PTSD‐related reactive aggression and perpetration‐related appetitive aggression can accelerate the ongoing cycle of violence (Elbert et al., 2018) mediated by distinct neural mechanisms (Moran et al., 2014).

The sense of omnipresent threat imposed by the fear/hunting network can fundamentally change beliefs about the world as a safe and benevolent place (Başoğlu et al., 2005), induce constant concerns about future threats (Weierstall et al., 2013), increase endorsement of violent means to end conflict (Vinck et al., 2007), anger and hostility (Orth & Wieland, 2006) and reduce openness to forgiveness (Nateghian et al., 2015; Witvliet et al., 2004) and reconciliation (Bayer et al., 2007). The belief of having lost control over fate (Schmidt et al., 2016) in a nonbenevolent and dangerous world (Biruski et al., 2014; Campbell & Vollhardt, 2014), where the future appears hopeless (Fehon et al., 2001) further increases the risk of violence. In case of ex‐combatants this means that facing social obstacles to reintegration when returning from armed group to civilian life likely further strengthens the hopelessness for a better future, renders investments in building a stable social integrity in vain and paves the way to yield to aggressive impulses.

### 1.2 Social obstacles to reintegration after armed group life

In Eastern DRC, about one quarter of civilians in population‐based surveys do not consider ex‐combatants welcome in their community (Humphreys, 2008) and many do not feel comfortable to share their daily life with them, including living in the same village, neighborhood, or household (66%), going to the same market, church, or school (46%), working together (56%), sharing a meal or drink (63%), or welcoming them into their family after they married a relative (40%, Vinck et al., 2008). Research has further shown that the direct exposure to stigma can provoke violent reactions: Leary et al., (2006) carried out a review including experimental, correlational, and longitudinal studies with varied samples worldwide and showed that interpersonal rejection can predict anger, the derogation of other people and, ultimately, aggressive behavior. Possible explanations of this relationship may be that rejection causes frustration and threatens self‐esteem and that aggression serves the regain of self‐control and social influence. This is further supported by results of a large‐scale study with 1,319 Spanish adolescents (age 11 to 16 years) which showed a relationship between the unfulfilled desire for more social recognition from peers with loneliness, dissatisfaction with life and violence (Buelga et al., 2008). Ex‐combatants who struggle to control their violent urges (cf. appetitive aggression) seem especially prone to increased mental health problems and externalizing behavior problems after stigmatization (Betancourt et al., 2013; Sommer et al., 2017) as it dismisses hopes of re‐establishing moral and social integrity in the new environment (cf., Shnabel & Nadler, 2008). A hindsight bias may further overemphasize positive aspects of armed group life (e.g., power, respect) whereas nontraumatic negative memories fade (e.g., insecurity, periods of hunger, restriction of autonomy). This not only constitutes an obstacle to retransition from soldier to civilian identity (Koebach et al., 2015; Wessels, 2016) but may further lead to ex‐combatants’ perceiving the rejoining of an armed group as an appealing option, especially in (post) conflict regions where military networks persist and rapidly fluctuate between active and passive states (Banholzer & Haer, 2014).

### 1.3 The present study

This study aims to investigate the association between the community's skepticism toward reintegration with ongoing violence perpetrated by ex‐combatants. In a representative community sample in Eastern DRC, we first examine (1) differences between ex‐combatants and other community members in trauma exposure, perpetration, mental health problems, perceived and experienced stigma, and skepticism toward reintegration of ex‐combatants; (2) the prevalence rates for the community's skeptical attitudes toward reintegration; and (3) the interplay of the community's skepticism toward reintegration with ex‐combatants’ perceived and experienced stigma, mental health problems, and ongoing violence controlled for trauma exposure. We hypothesize that the community's skepticism toward reintegration in combination with ex‐combatants’ perceived and recently experienced stigma predicts their mental health problems and ongoing violence in addition to trauma exposure.

## 2 MATERIALS AND METHODS

### 2.1 Procedure

Between September 2018 and May 2019, a representative sample from six communities with 687 households and 1946 adult inhabitants were interviewed in the Kivu regions, Eastern DRC. This survey represents the baseline of a longitudinal randomized controlled trial with three follow‐up timepoints (post, three and six months) measuring the effectiveness of a community intervention to reduce stigmatizing attitudes toward trauma survivors including ex‐combatants and increase outreach of mental health services after trauma. Hereafter, only baseline data were analyzed. However, information about participants who disclosed armed group involvement not at baseline but at a later timepoint was used to determine the group of ex‐combatants. For each community, we calculated the sample size by controlling for sex and age distributions (male versus female, 16 to 36 years versus 37 to 57 years versus over 57 years) with a 5% margin of error and 95% CI. Diagnostic interviewers selected participants in door‐to‐door visits by blind and random drawing of folded papers labeled with the respective sex and age category. If selected participants were absent, interviewers organized later appointments and if multiple inhabitants of one household were eligible (within the chosen category), the person who chose a paper marked with a black‐cross out of a subset of empty papers was interviewed. In isolated cases, residents denied participation due to lack of time (because of work or school responsibilities) a further paper was drawn. After visiting all households, a subsample of community members was invited for interviews nonrandomly, in order to obtain a representative sample size. This recruitment was facilitated by individuals with respected local authority. After randomization and indication of availability, individuals were informed about the study procedure. None of these individuals declined participation. Twenty‐one trained local psychologists conducted clinical interviews (1.5 to 2.5 hr) under supervision by the authors on site. Questionnaires were translated from English to Kiswahili and back by two native Congolese interpreters and discrepancies discussed with two of the authors. Participants received light refreshment and small financial compensation (1.000 CDF, ca. 0.60$). Interviewers double rated forty‐two assessments for the calculation of interrater reliability. The study was approved by the ethic commission of the University of Konstanz (31/2016) and the Social Fund of the DRC.

### 2.2 Participants

A total of 1,066 community members participated in the study. 13% (*n* = 137) indicated a history of armed group involvement, whereby only a minority of them were female (6%, *n* = 8). As female and male recruits present with essential characteristic disparities, this paper focuses on male ex‐combatants only. The final sample contains 1,058 participants with 129 (12%) ex‐combatants. Participants were almost equally distributed in regard to sex (52%, *n* = 545, women), on average 36 years old (*SD* = 16.7, range 16–91 years), mostly in a partnership (62%, *n* = 652), had on average four children (*SD* = 3.5, range 0–30) and indicated on average five years of education (*SD* = 4.5, range 0–19) and low financial wealth (*M* = 2, *SD* = 0.8, range 1–5). All were Congolese nationals. The most common native languages were Kiswahili (49%, *n* = 516) and Hunde (27%, *n* = 284; 25%, *n* = 261, indicated another language). One fifth (20%, *n* = 208) spoke more than one language. Almost everyone was religious (98%, *n* = 1,041, of which 98% were Christians, 1% Islam, and 1% others). About half of the participant migrated into the communities (52%, *n* = 547), for example, due to security issues (incl. loss of home, *N* = 185, 34%), marriage (*N* = 221, 40%), stigmatization in their last communities (*N* = 45, 8%), or other reasons (e.g., search for work or family reunion, *N* = 96, 18%). Ex‐combatants were younger than other community members (*t*(216) = 2.9, *p* = .004), more often in a partnership (χ^2^(1) = 7.9, *p* = .005) and more educated (*t*(1,056) = −7.0, *p* < .001). The groups did not differ in the number of children, immigration status (born in village versus immigrated), and wealth (*p* > .05).

### 2.3 Assessment

We assessed demographic information on sex, age, education, partnership, number of children, immigration, financial wealth, armed group history as well as trauma exposure, perpetration, experienced and perceived stigma (operationalized as actual experiences of stigma in the community and its internal effects including feelings of shame and the perceived lack of social acknowledgement), mental health problems, and skepticism toward reintegration of ex‐combatants.

#### 2.3.1 Trauma exposure, perpetration, and experienced stigma

The frequency of different traumatic event types, experienced stigma, and perpetration was assessed with the 41‐item version of the Threats to Human Life Scale (THL, scale can be obtained from the authors, , in prep.). Participants indicate the experience (yes/no) of recent (last three months) and lifetime (prior to the last three months) threat types to physical (18 item, e.g., suffocation) and social integrity (8 items, e.g., social exclusion) and types of perpetration (15 items, e.g., physical fighting, sexual assault). For each event, the scale asks for the aggressor (family member/person of trust, community member, stranger/organized violence, and non‐man‐made reason) and ex‐combatants additionally indicated if they believed to have experienced social threats because of their armed group history. For perpetrated events, participants were asked against whom the violence was directed (family member/person of trust, community member, and stranger/organized violence). The subscale sum scores are based on event occurrence (lifetime, recent) and context (family, community, stranger/organized violence, non‐man‐made; physical threats/trauma exposure: range 0–90, social threats/experienced stigma: range 0–15, perpetration: range 0–60).

#### 2.3.2 Mental health problems

The PTSD Symptom Scale‐Interview for DSM‐5 (PSS‐I‐5, Foa & Capaldi, 2013; Foa et al., 2016) was used to assess PTSD symptom severity in the last month. Participants indicated the presence of 20 symptoms from 0 *(Not at all)* to 4 *(6 or more times a week/severe)* in relation to an index trauma. A sum score indicated PTSD symptom severity (range 0–80) and diagnosis was calculated following the manual. Prior research demonstrated the scale's applicability in African countries (Ertl et al., 2011) including Eastern DRC (Robjant et al., 2019). Internal consistency (α = 0.95) and interrater reliability (IRR = 0.98) were excellent.

We assessed depressive symptoms in the last two weeks with the 9‐item Patient Health Questionnaire (PHQ‐9; Kroenke et al., 2001). Participants rated item from 0 *(Not at all)* to 3 (*Nearly every day*). A sum score (range 0–27) indicated depression symptom severity and diagnosis of Major Depression was ascertained according to DSM‐5 (The American Psychiatric Association, 2013). The scale has been applied all over the world including Eastern DRC (Koebach et al., 2015). Reliability measures were excellent (α = 0.85, IRR = 0.98).

Attraction to violence was assessed among ex‐combatants with the Appetitive Aggression Scale (AAS; Weierstall & Elbert, 2011). Participants rated 15 items from 0 *(Disagree)* to 4 *(Agree)*, whereby the sum score indicates a stronger desire for violence (range 0–60). The scale has been used in DRC among male and female ex‐combatants (Koebach et al., 2015; Robjant et al., 2019). Cronbach's Alpha was excellent (α = 0.90). It was not possible to calculate interrater reliability as interraters were randomly allocated and, due to the assessment of AAS only among ex‐combatants, not enough data were collected.

#### 2.3.3 Perceived stigma

Feelings of shame were measured with the 14‐item *Shame Variability Questionnaire* (SVQ, Brown et al., 2001). Participants indicated their shame on a scale from 0 *(Not at all/I did not feel this way)* to 4 *(Completely/I felt this very strongly)* for a time in the last four months when they felt the most shame or worst about themselves. A sum score was calculated (after recoding two inverse items) indicating feelings of shame (range 0–56). The scale was successfully applied in a sample with Africans (Stotz et al., 2015). Reliability measures were good (α = 0.84, IRR = 0.96).

We administered the “general disapproval” subscale of the *Social Acknowledgement Questionnaire* (SAQ, Maercker & Mueller, 2004) to assess the perceived lack of general social acknowledgement as a trauma survivor. This subscale demonstrated the strongest correlates with PTSD compared to the “recognition” and “family/friends disapproval” subscales (Jones et al., 2006; Mueller et al., 2009; Wagner et al., 2012). Participants indicated their perception of acknowledgement as trauma survivor from 0 *(I do not agree at all)* to 3 *(I completely agree)* on five items with reference to a traumatic event after which they needed social support. The sum score indicated the perceived lack of general social acknowledgement (range 0–15). Reliability measures were satisfying (α = 0.69, IRR = 0.95).

#### 2.3.4 Skeptical attitudes toward reintegration of ex‐combatants

Skepticism toward reintegration of ex‐combatants was assessed with the *Social Reconstruction* *Scale* (SoRS; Ajduković et al., 2011). Originally developed in postgenocide Bosnia Herzegovina to measure Croats’ and Serbs’ readiness to reconcile, we adapted the scale to measure the social fabric between ex‐combatants and other community members. On 19 items (two items were crossed out due to nonapplicability for the context of DRC), participants rated their attitudes toward reapproach, trust and need for apology from 0 *(Disagree)* to 4 *(Agree)*. To our knowledge, the scale has not been used in a comparable setting yet. The sum score indicates skeptical attitudes toward reintegration of ex‐combatants (range 0–76). Internal consistency and interrater reliability were satisfying (α = 0.76, IRR = 0.94).

### 2.4 Statistical analyses

SPSS 26 (IBM, 2019) was used to carry out statistical analysis. Missing values were mean imputed for variables that comprised up to 5% missing (Tsikriktsis, 2005). Differences between ex‐combatants and other community members in trauma exposure, perpetration, mental health problems, perceived and experienced stigma, and skepticism toward reintegration of ex‐combatants were explored with the Mann–Whitney U test. Conditional process analysis (Model 59, Hayes, 2017) combining mediation and moderation regression analysis was applied in the sample of ex‐combatants to test the association of the community's skepticism toward reintegration with ongoing violence under consideration of perceived stigma and mental health problems as mediators and recently experienced stigma as moderator. The community's skepticism toward reintegration was assessed as grand group mean of SoRS per village among individuals who denied participation or abduction into an armed group. Perceived stigma is calculated as sum score of the z‐transformed SAQ and SVQ, recently experienced stigma as ex‐combatants’ THL recent threats to social integrity and mental health problems as sum score of the z‐transformed PSS‐I, PHQ‐9, and AAS (which all showed associations with ongoing violence in previous studies, Nandi et al., 2017; Taft et al., 2009). Ongoing violence represents the THL recent perpetration score. Due to zero inflation and few extreme values, the score was added as ordinal variable based on its percentiles. The analysis was controlled for ex‐combatants’ lifetime trauma exposure (THL threats to physical integrity). Assumptions of linearity, nonmulticollinearity, independence, and normality of residuals were met except for the third regression model including ongoing violence as outcome for which bootstrapped standard errors and confidence intervals were calculated due to non‐normal distribution of residuals. Results are reported including outliers as calculation with and without outliers indicated no substantial differences.

## 3 RESULTS

### 3.1 Armed group history and social disclosure

Ex‐combatants enrolled in armed groups at an average age of 18 years (*SD* = 6.5, range: 0–30, note that some ex‐combatants are born and raised within armed groups). The majority were part of military missions (70%, *n* = 89, missing *n* = 10) mostly with combat experience (72%, *n* = 64 indicated up to 10 times, 25%, *n* = 22 multiple times and 2%, *n* = 2 no fighting, missing *n* = 1). At baseline, 61 (85%, missing *n* = 57) ex‐combatants indicated that their armed group history was socially disclosed: In 16 (27%) of the cases, confidents were the only persons who were informed, whereas 44 (73%) stated that neighbors or the whole community also knew about their past. Reluctance to disclose was likewise reflected in this study as 57 (44%) ex‐combatants had not disclosed their armed group history to interviewers at baseline but at a later timepoint of the research trial. The main reasons reported to interviewers were initial skepticism about confidentiality and fear of disclosure to and persecution by the community or even prosecution by the police.

### 3.2 Differences between ex‐combatants and other community members

Table 1 shows differences between ex‐combatants and other community members in regard to trauma exposure, perpetration, mental health problems, perceived and experienced stigma, and skepticism toward reintegration of ex‐combatants. Almost one third (28%, *n* = 301) fulfilled the diagnostic criteria of PTSD with higher prevalence rates among ex‐combatants (47%, *n* = 60) than other community members (26%, *n* = 241). Ninety‐two (9%) presented with a diagnosis of major depression, whereby ex‐combatants (9%, *n* = 11) and other community members (9%, *n* = 81) showed comparable prevalence rates. Almost all ex‐combatants approved at least one item of appetitive aggressive (96%, *n* = 126).

**TABLE 1:** Trauma, perpetration, mental health problems, and social outcomes presented as means followed by standard deviations and ranges

	Total (*N* = 1,058)	No ex‐combatants (*n* = 929)	Ex‐combatants (*n* = 129)	Cohen's *d*
Exposure to violence (THL, threats to physical integrity)				
Lifetime exposure	9.0 [3.4; 0–18]	8.7 [3.4; 0–18]	11.0 [3.2; 2–17]	0.45^***^
Recent exposure by…				
…family/person of trust	0.6 [1.0; 0–8]	0.6 [0.9; 0–8]	0.7 [1.0; 0–5]	0.09
…other community member	1.2 [1.4; 0–8]	1.1 [1.4; 0.8]	1.7 [1.7; 0–8]	0.25^***^
…strangers/organized violence	0.6 [1.0; 0–10]	0.6 [1.0; 0–10]	0.9 [1.4; 0–8]	0.19^**^
…non‐man‐made reason	1.6 [1.6; 0–8]	1.6 [1.6; 0–8]	1.8 [1.6; 0–6]	0.11
Perpetration of violence (THL, perpetration)				
Lifetime perpetration	2.1 [2.2; 0–13]	1.8 [1.9; 0–12]	4.0 [3.2; 0–13]	0.51^***^
Recent perpetration against…				
…family/person of trust	0.3 [0.8; 0–7]	0.3 [0.8; 0–6]	0.4 [1.0; 0–7]	0.04
…other community member	0.3 [0.7; 0–5]	0.3 [0.7; 0–5]	0.5 [1.0; 0–4]	0.15*
…strangers/organized violence	0.0 [0.3; 0–6]	0.0 [0.2; 0–1]	0.2 [0.7; 0–6]	0.18^**^
Mental health problems				
PTSD symptom severity (PSS‐I−5)	9.6 [11.8; 0–62]	8.9 [11.4; 0–62]	14.2 [13.2; 0–57]	0.30^***^
Depression symptom severity (PHQ−9)	6.8 [5.1; –27]	6.7 [5.1; 0–26]	7.3 [5.1; 0–27]	0.09
Appetitive aggression (AAS)	NA	NA	14.4 [13.3; 0–59]^a^	NA
Experienced stigma (THL, threats to social integrity)^b^				
Lifetime exposure	3.5 [2.0; 0–8]	3.4 [2.0; 0–8]	4.2 [2.0; 0–8]	0.26^***^
Recent exposure	1.6 [1.4; 0–7]	1.6 [1.4; 0–7]	1.7 [1.4; 0–6]	0.07
Felt stigma				
Feelings of shame (SVQ)	29.2 [12.0; 0–56]	28.9 [11.9; 0–56]	30.9 [12.4; 0–56]	0.10
Perceived lack of general social acknowledgment (SAQ)	5.2 [3.9; 0–15]	5.2 [3.9; 0–15]	5.8 [4.2; 0–15]	0.09
Skepticism toward reconstruction with ex‐combatants (SoRS)				
No reapproach	6.0 [5.4; 0–27]	6.4 [5.5; 0–27]	3.4 [4.0; 0–21]	0.40^***^
Mistrust	16.5 [6.8; 0–34]	17.0 [6.7; 0–34]	13.3 [6.7; 0–32]	0.35^***^
Need for apology	10.0 [2.5; 0–12]	10.1 [2.5; 0–12]	9.8 [2.8; 0–12]	0.04

^a^ AAS was assessed among 69 ex‐combatants.

^b^ 45% ex‐combatants (*n* = 33 of *N* = 74) stated to have experienced at least one social threat because of their armed group history.

* *p* < .05, **p < .01, ***p < .001.

### 3.3 Prevalence rates for the community's skepticism toward reintegration of ex‐combatants

Prevalence rates for the community's skeptical attitudes toward reintegration of ex‐combatants are presented in Table 2. There were differences between communities, *X* ^2^ (5, *N* = 929) = 28.8, *p* < .001, whereby one community in particular presented with less beliefs in reintegration than others in post hoc tests. The percentage of ex‐combatants in this community (18%, *n* = 32 of 176 inhabitants) was higher than in the others (11%, range: 5%–17%, 97 of 882 inhabitants, *X* ^2^ (1, *N* = 1,058) = 7.1, *p* = .008).

**TABLE 2:** Affirmations^a^ for the community's skepticism toward reintegration of ex‐combatants presented as percentages followed by frequencies (*n* = 929)

			% (*n*)
NRA^b^	1	I believe that working on common goals is the best way to restore trust between the community and ex‐combatants.	61 (565)
	2	The state should equally care for everyone affected by war regardless of the side they belonged to, that is, also for ex‐combatants.	77 (716)
	5	I think it's important for our children to cooperate with children of ex‐combatants.	69 (644)
	6	I also sympathize with ex‐combatants who have lost someone.	86 (796)
	9	I feel sorry that ex‐combatants also lost their houses.	81 (756)
	13	I empathize with ex‐combatants who have done nothing wrong and suffer because of the wrongdoings done by members of their armed group.	87 (809)
	17	I believe that ex‐combatants also suffered during the war.	93 (865)
			**avg. 79**
MT	3	I believe in the principle “an eye for an eye and a tooth for a tooth.”	10 (90)
	4	I think that the trust between the community and ex‐combatants has been lost forever.	56 (520)
	7	Only those who have lost someone are entitled to say if it's all right for the community and ex‐combatants to start to cooperate.	15 (143)
	10	I think that it is impossible to overcome injuries that were inflicted in the last war between the community and ex‐combatants.	33 (305)
	12	I am not ready to cooperate with ex‐combatants even if my community asked me to do so.	30 (278)
	14	I do not like it when members of my community do business with ex‐combatants.	29 (268)
	15	I can be close with some ex‐combatants, but generally I do not trust them.	72 (673)
	18	I do not trust ex‐combatants.	63 (582)
	19	Community members should always be cautious in relations with ex‐combatants.	82 (762)
			**avg. 43**
AP	8	I would like ex‐combatants to show remorse for our victims.	85 (791)
	11	For better relations between the community and ex‐combatants it would be enough if they paid tribute to our victims.	85 (786)
	16	For me it is important that ex‐combatants apologize.	86 (803)
			**avg. 85**

Abbreviations: AP, need for apology; MT, mistrust; NRA, no reapproach.

^a^ Likert scale responses range from 0 to 4; displayed percentages represent item affirmation of 3 or 4.

^b^ Items recoded for subscale score.

### 3.4 Interplay between the community's skepticism toward reintegration and ex‐combatants’ perceived and recently experienced stigma, mental health problems, and ongoing violence

**FIGURE 1:**
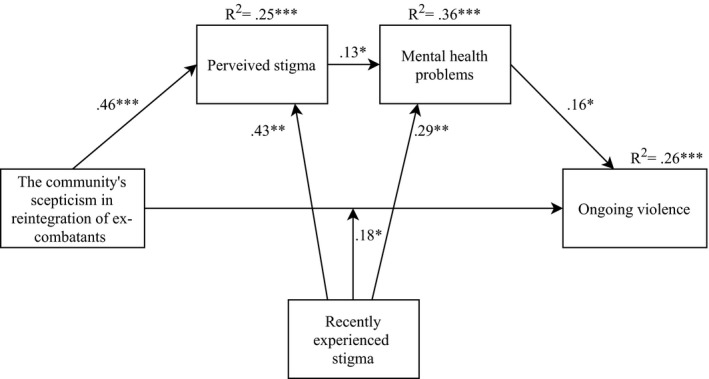
Conditional process analysis among male ex‐combatants on the relationship of the community's skepticism in reintegration (SoRS among non‐ex‐combatants) with ex‐combatants’ perceived stigma (SVQ, SAQ), mental health problems (PSSI‐5, PHQ‐9, and AAS), and ongoing violence (THL recent perpetration) moderated by recently experienced stigma (THL recent threats to social integrity) and controlled for lifetime trauma (THL threats to physical integrity, *N* = 129). Standardized coefficients are displayed for significant association paths. Only significant associations are shown

The relation between the community's skepticism toward reintegration and ex‐combatants’ perceived and recently experienced stigma, mental health problems, and ongoing violence controlled for trauma exposure is presented in Figure 1. Perceived stigma was predicted by the community's skepticism toward reintegration of ex‐combatants, *ß* = 0.46, *SE* = 0.12, *p* < .001 and recently experienced stigma, *ß* = 0.43, *SE* = 0.15, *p* = .004. Mental health problems were predicted by perceived stigma, *ß* = 0.13, *SE* = 0.06, *p* = .031, recently experienced stigma, *ß* = 0.29, *SE* = 0.10, *p* = .006, and trauma exposure, *ß* = 0.45, *SE* = 0.10, *p* < .001. Ex‐combatants’ ongoing violence was predicted by high recently experienced stigma, *ß* = 0.28, *SE* = 0.11, *p* = .009, but neither by low or moderate experienced stigma (moderation effect, *ß* = 0.18, Boot*SE* = 0.07, 95% BootCI = [0.03, 0.32]), and by mental health problems, *ß* = 0.16, Boot*SE* = 0.07, 95% BootCI = [0.03, 0.32]. Conditional analyses revealed no indirect effects of the community's skepticism in reintegration on ex‐combatants’ ongoing violence via perceived stigma and mental health problems (*p* > .05).

## 4 DISCUSSION

This study showed that ongoing violence perpetrated by male ex‐combatants in Eastern DRC is not only enhanced by mental health problems but also by the social environment. Specifically, implicit beliefs and overt interaction with the community, specifically their skepticism in reintegration of the demobilized and stigmatization against them as well as ex‐combatants’ feelings of being stigmatized. Although all community members presented with a high level of trauma exposure, ex‐combatants constitute a group with a particularly destructive mixture of lifetime trauma and perpetration. As consequence to their war trauma history, appetitive aggression is highly prevalent and PTSD symptoms were more severe than the average in the community.

### 4.1 Trauma exposure and perpetration

Twelve percent of participants who admitted an armed group history also presented with significantly more perpetration and lifetime trauma (cf., Elbert et al., 2013) than other community members. They further indicated more recent conflict with community members and strangers/organized violence (i.e., both higher victimization by and more perpetration against them). There was no difference in self‐reported violence against the family/persons of trust. Prior research has shown that combat‐related trauma is not associated with violence against children but intimate partner violence (cf., Nandi et al., 2017)—though no direct comparison with other members of the community was made. However, countries in Sub‐Saharan Africa present with one of the highest prevalence rates of violence against women (Hoeffler, 2018), in particular Eastern DRC (57% of women, The Demographic Health Survey, 2014). Beyond combat‐related trauma, many other factors account for violence against female partners including unequal gender norms and “spread” of political to civilian violence (Kelly et al., 2018, 2019), which may explain the comparable prevalence rates regarding perpetration in the private realm.

### 4.2 The community's skepticism toward reintegration and ex‐combatants’ perceived and recently experienced stigma

Ex‐combatants showed less skepticism than their social environment regarding social reapproach and the restoration of trust. This is in line with prior research that indicated high levels of rejecting attitudes among the general population (Humphreys, 2008; Vinck et al., 2008). However, both believed that remorse and apologies by ex‐combatants were prerequisites. Showing remorse may be regarded as an indicator that psyche and morality were not irreversibly changed while being in the armed group, as is commonly believed (Harvard Humanitarian Initiative, 2013), but that ex‐combatants are willing (and capable) to readjust to peaceful conditions and to re‐establish their moral and social integrity (cf., Shnabel & Nadler, 2008). Whereas ex‐combatants indicated more lifetime threats to social integrity than other community members, there was no difference in recently experienced social threats or perceived stigmatization (shame, perceived lack of social acknowledgement as trauma survivor). However, almost half of them believed to have experienced at least one social threat because of their armed group history.

### 4.3 Understanding the path to mental health problems and the continuation of violence

Beyond the impact of trauma, recently experienced stigma was found to be associated with ex‐combatants’ perceived stigma, mental health problems, and continuation of violence. This is in line with prior research showing an association between stigmatization and psychopathology (cf. Betancourt et al., 2013; Sommer et al., 2017) and aggression (cf., Buelga et al., 2008; Leary et al., 2006). In our study, experienced stigma not only directly predicted ex‐combatants’ perceived stigma and mental health problems but also moderated the impact of the community's skeptical attitudes toward reintegration on their ongoing violence. This ongoing violence which can be observed in public places either against members from the community or strangers/organized violence can further lower the community's belief that ex‐combatants are willing (and capable) to adapt to non (or less) violent civilian life and increase anxiety, mistrust and caveats to integration, which ex‐combatants likely experience as stigma and obstacles to reintegration. Ultimately, a downward spiral seems to arise that accelerates the cycles of violence: Ex‐combatants who live in a social environment that holds strong rejecting attitudes toward former recruits, who encounter social threats (which they often attribute to their armed group history), and who perceive this stigma doubt that reintegration and re‐establishment of their social reputation are possible and in consequence likely feel lonely and dissatisfied with their “new” civilian life are likely less successful in minimizing trauma‐ and combat‐related aggressive tendencies, which again decreases others’ openness to reapproach and trust.

### 4.4 Implications

Beyond traumatic experiences, the community's rejecting attitudes and enacted stigmatization account for mental health problems and ongoing violence among ex‐combatants. Whereas caution toward and rejection of ex‐combatants are presumably designed as protection and shall increase the community's security, this study shows that it can predict the opposite effect and rather increase the risk of ex‐combatants’ further perpetration. Effective reintegration programs should therefore address ex‐combatants’ psychopathology (e.g., PTSD, depression, appetitive aggression, and substance abuse) including aggressive tendencies (e.g., Narrative Exposure Therapy for Forensic Offender Rehabilitation, FORNET, original paper Elbert et al., 2012; development Robjant et al., 2019; Koebach et al., 2021) as well as social tensions. Social approaches have shown promising results; however, they often either required intense individualized therapeutic guidance (e.g., multisystemic therapy, Limbos et al., 2007) or addressed general and not community‐specific discriminatory attitudes and behavior toward particular groups (e.g., radio edutainment, Iqbal & Bilali, 2017). Stringent scientific evaluation is often lacking particularly for large‐scale programs. To ultimately break the cycles of violence, a shift toward an integrated approach is indicated that addresses mental health problems and perpetration both at the individual and at the community level (e.g., Robjant et al., 2020b).

### 4.5 Limitations

Limitations include the constrain on explicit measures, which may imply a bias of social desirability. All data were collected in rural areas where insecurity was somehow calculable. Moreover, female ex‐combatants who make up to 48% of armed groups in Eastern DRC (Johnson et al., 2010; Mels et al., 2009) were underrepresented in our study and due to the small sample size excluded from the analyses. Rejecting attitudes and behavior toward women with a history of armed group membership needs further investigation. Finally, generalizability of the results of the path analysis may be restricted due to small sample size of ex‐combatants.

## 5 CONCLUSION

Ex‐combatants in Eastern DRC face multiple obstacles to reintegration after returning from the battlefield and present with the risk of fueling the cycles of violence in the community. This study demonstrated that, in addition to lifetime trauma, social adversities account for ex‐combatants’ mental health problems and continuation of violence in civilian life. Comprehensive reintegration programs should address trauma‐ and combat‐related mental health and behavior problems both at the individual and the community level.

## CONFLICT OF INTEREST

The authors have no conflict of interest to declare.

## AUTHOR CONTRIBUTIONS

**Sabine Schmitt** involved in conceptualization, methodology, formal analysis, investigation, data curation, writing–original draft, review and editing, visualization, and project coordination. **Katy Robjant** involved in investigation, writing–review and editing, project management, and funding acquisition. **Anke Koebach** involved in conceptualization, methodology, supervision, writing–review and editing, and funding acquisition.

### PEER REVIEW

The peer review history for this article is available at https://publons.com/publon/10.1002/brb3.2156.

## Data Availability

The data that support the findings of this study are available from the corresponding author upon reasonable request.
